# Silver Nanoparticles Containing Fucoidan Synthesized by Green Method Have Anti-*Trypanosoma cruzi* Activity

**DOI:** 10.3390/nano12122059

**Published:** 2022-06-15

**Authors:** Adriana Oliveira Souza, Johny Wysllas de Freitas Oliveira, Claudia Jéssica Gonsalves Moreno, Mayra Jane Campos de Medeiros, Marília Medeiros Fernandes-Negreiros, Flavia Roberta Monteiro Souza, Daniel Lima Pontes, Marcelo Sousa Silva, Hugo Alexandre Oliveira Rocha

**Affiliations:** 1 Laboratório de Biotecnologia de Polímeros Naturais (BIOPOL), Departamento de Bioquímica, Centro de Biociências, Universidade Federal do Rio Grande do Norte (UFRN), Natal 59078-970, RN, Brazil; adri.positivo95@gmail.com (A.O.S.); marilia_negreiros16@yahoo.com.br (M.M.F.-N.); flavia.shaupe@gmail.com (F.R.M.S.); 2 Laboratório de Imunoparasitologia, Departamento de Análises Clínicas e Toxicológicas, Centro de Ciências da Saúde, Universidade Federal do Rio Grande do Norte, Natal 59012-570, RN, Brazil; johny3355@hotmail.com (J.W.d.F.O.); claudia.mrn1@gmail.com (C.J.G.M.); mssilva@ihmt.unl.pt (M.S.S.); 3 Laboratório de Química de Coordenação e Polímeros (LQCPol), Departamento de Química, Instituto de Química, Universidade Federal do Rio Grande do Norte (UFRN), Natal 59078-970, RN, Brazil; mayarajane20049@hotmail.com (M.J.C.d.M.); pontesdl@yahoo.com (D.L.P.); 4 Global Health and Tropical Medicine, Institute of Hygiene and Tropical Medicine, University of Nova Lisboa, 1099-085 Lisbon, Portugal

**Keywords:** fucoidan, chagas disease, brown seaweed, anti-*Trypanosoma cruzi* activity

## Abstract

The brown seaweed *Spatoglossum schröederi* synthesizes three bioactive fucoidans, the most abundant of which is fucan A. This fucoidan was extracted and its identity was confirmed by chemical analysis, Fourier-transform infrared spectroscopy (FTIR), and agarose gel electrophoresis. Thereafter, silver nanoparticles containing fucan A (AgFuc) were produced using an environmentally friendly synthesis method. AgFuc synthesis was analyzed via UV–vis spectroscopy and FTIR, which confirmed the presence of both silver and fucan A in the AgFuc product. Dynamic light scattering (DLS), X-ray diffraction, scanning electron microscopy, and atomic force microscopy revealed that the AgFuc particles were ~180.0 nm in size and spherical in shape. DLS further demonstrated that AgFuc was stable for five months. Coupled plasma optical emission spectrometry showed that the AgFuc particles contained 5% silver and 95% sugar. AgFuc was shown to be more effective in inhibiting the ability of parasites to reduce MTT than fucan A or silver, regardless of treatment time. In addition, AgFuc induced the death of ~60% of parasites by necrosis and ~17% by apoptosis. Therefore, AgFuc induces damage to the parasites’ mitochondria, which suggests that it is an anti-*Trypanosoma cruzi* agent. This is the first study to analyze silver nanoparticles containing fucan as an anti-Trypanosoma cruzi agent. Our data indicate that AgFuc nanoparticles have potential therapeutic applications, which should be determined via preclinical in vitro and in vivo studies.

## 1. Introduction

Chagas disease (CD), known as American trypanosomiasis, is a potentially life-threatening zoonotic illness that was discovered and characterized by the Brazilian researcher Carlos Chagas in 1909. The natural mechanism of transmission is through Triatomineos insects, which can transmit the etiological agent *Trypanosoma cruzi* to a vertebrate host during the blood meal. The etiological agent of CD is a parasitic flagellate with a heteroxenous life cycle, which has more than one host and more than one evolutionary life form. According to the morphological and physiological changes in the host, the parasite can be found in three main evolutionary forms, namely, the epimastigote, trypomastigote, and intracellular amastigote [1,2,3]. CD is considered a neglected tropical disease, and is endemic in 21 continental Latin American countries [4]. In addition, CD has been described in other non-endemic countries, such as the United States of America, Canada, many European countries, and several African, Eastern Mediterranean, and Western Pacific countries [4,5]. An estimated 6 to 7 million people worldwide are infected with *T. cruzi* [4]. Infected patients may experience symptoms such as hepatosplenomegaly, lymphadenopathy, and facial edema in the acute phase of the disease. However, the chronic phase is usually asymptomatic, making diagnosis difficult and resulting in cardiovascular [4,6,7] and gastrointestinal complications [8].

The treatment of CD is based on the use of nifurtimox and benznidazole, which are nitroheterocyclic agents with a mechanism of action consisting of inducing the formation of reactive oxygen species, such as superoxide radicals and hydrogen peroxide. These reactive oxygen species then induce oxidative damage to the parasite, leading to its death. However, patients using these drugs may experience several serious side effects [9,10], such as liver and pancreatic damage, as well as skin irritation [11]. When CD is diagnosed and properly treated, at least 5% of cases per year treated in the acute and indeterminate stages reduce transmission and bring economic and health benefits [12], showing the importance of the need for improved diagnostics and access to safe and effective treatment. In view of this scenario, it is necessary to search for new compounds that can be used as anti-*T. cruzi* drugs that are inexpensive, effective, and less toxic to the patient.

Fucoidans are sulfated polysaccharides that have various activities [13]. Moreover, they can be used as raw materials for the synthesis of biomaterials and nanomaterials [14], thereby further diversifying their applications. Despite this, we identified only one paper that investigated fucoidans with anti-*T. cruzi* activity. The paper in question demonstrated that a fucoidan obtained from *Lessonia* sp. seaweed presented cytotoxicity against *T. cruzi* epimastigotes at a concentration of 250 μg/mL [15].

One way to enhance the activity of fucoidan is to insert it into a nanoparticle. Recently, we synthesized silver nanoparticles with a fucoidan-rich fraction isolated from the *Spatoglossum schröederi* seaweed and found that this nanoparticle had antiproliferative activity against murine melanoma cells that was three times greater than that of the native fucoidan [16]. However, the effects of this fucoidan against *T. cruzi* have yet to be evaluated.

The production of silver nanoparticles enriched with sulfated polysaccharides isolated from seaweed, including fucoidans, can be achieved using a “green” method which is inexpensive, effective, and environmentally friendly [17]. The brown seaweed *S. schröederi* synthesizes three types of bioactive fucoidans, the most abundant of which is named fucan A. Therefore, in this study, we aimed to obtain fucan A from *S. schröederi* seaweed, synthesize nanoparticles containing this fucoidan, and characterize it structurally. In addition, we aimed at a possible application for this nanoparticle, in this case as a possible anti-trypanosome agent.

## 2. Materials and Methods

### 2.1. Materials

Coomassie brilliant blue R-250 (27816), resazurin (R7017), silver nitrate—AgNO3 (85193), 1,3-diaminopropane (D23602), cresol red (114472), chondroitin sulfate (C9819), dermatan sulfate (C3788), heparan sulfate (H7640), D-galactose (G0750), D-glucose (G8270), L-arabinose (A3256), D-mannose (M6020), L-fucose (F2252), D-fructose (F0127), D-xylose (X1500), D-glucosamine (G4875), D-glucuronic acid (G5269), D-mannuronic acid (SMB00280), D-rhamnose (R3875), bovine serum albumin—BSA (A7030), and the components of the cell culture liver infusion tryptose (LIT) media (61724), namely, dextrose, hemin (51280), tryptose (70937), and liver infusion broth (CM0077B), were all purchased from Sigma (St. Louis, MO, USA). Sterile fetal bovine serum—FBS (F0063) was purchased from Cultilab (Campinas, SP, Brazil). Penicillin and streptomycin (15070063) were obtained from Thermo Fisher Scientific (Waltham, MA, USA). All other solvents and chemical products used in this study were of analytical grade purity.

Y strains of the *T. cruzi* parasite were provided by Aline Rimoldi Ribeiro from the State University of Campinas—UNICAMP, Campinas-SP, Brazil, which were isolated from human cases. They were grown in liver infusion tryptose (LIT) medium supplemented with 10% inactivated FBS and 5% streptomycin/penicillin (100 UI/mL). The cultures were maintained at 27 °C for the epimastigote forms of *T. cruzi* [18].

### 2.2. Seaweed Collection

The brown seaweed *S. schröederi* was collected in the city of Nísia Floresta (05°58′23″ S–35°04′57” W) on the southern coast of the state of Rio Grande do Norte, Brazil, in March 2019. After collection, the seaweed was transported to the Laboratório de Biotecnologia de Polímeros Naturais (BIOPOL) of the Departamento de Bioquímica, Universidade Federal do Rio Grande do Norte (UFRN) for the removal of epiphytic species, sediment, and encrusted organisms and subsequent extraction of fucoidans. The seaweed was identified according to its morphology [19] by Dr. Valquiria Pereira, Federal University of Juiz de Fora-MG, Brazil. A voucher specimen was deposited in the UFRN Herbarium of the Institute of Biosciences, Federal University of Rio Grande do Norte, under the registration code UFRN25521. Material collection occurred under the authorization of the Brazilian National System of Management of Genetic Heritage and Associated Traditional Knowledge SISGEN n° A0D4240.2.1.1.

### 2.3. Extraction of Fucoidans

Extraction of fucoidans was performed as described by Rocha et al. [20]. In order to obtain the fucoidan-rich extract, the seaweed was dried immediately after collection at 50 °C under ventilation and then ground in a blender. The samples were then treated with ethanol to eliminate lipids and pigments. Afterwards, 100 g of defatted, dried, and powdered seaweed was suspended in 500 mL of 0.25 M NaCl and the pH was adjusted to 8.0 using NaOH. Subsequently, a mixture of alkaline proteases (Prolav 750—Prozyn Biosolutions, São Paulo, SP, Brazil) at 15 mg/g was added to the powdered seaweed and proteolytic digestion was conducted for 18 h at 60 °C. The fucoidan-rich extract was obtained after filtration and was subjected to fractionation as described by Almeida-Lima et al. [20]. Briefly, 0.5 volumes of propanone (4 °C) were added to the fucoidan-rich extract under gentle agitation and then incubated for 24 h. The samples were then centrifuged at 10,000× *g* for 20 min, dried, and kept in the dark until further use. The fractionation was repeated by adding 0.6, 0.7, 0.9, 1.1, 1.3, and 2.0 volumes of propanone to the supernatant. Based on the propanone volumes used, the fractions were named F0.5v, F0.6v, F0.7v, F0.9v, F1.1v, F1.3v, and F2.0v, respectively.

### 2.4. Agarose Gel Electrophoresis

All precipitate fractions obtained were analyzed by agarose gel electrophoresis as previously described [21]. Samples (50 μg) were applied to a 0.6% (*w*/*v*) agarose gel and ran for 1 h at 110 V in 0.05 M 1,3 diamino propane/acetate (PDA) buffer, pH 9.0. Afterwards, the fucoidans were fixed in the gel with 0.1% CTV solution for 12 h. Following fixation, the gel was dried under airflow and stained with 0.1% (*w*/*v*) toluidine blue in a solution of acetic acid:ethanol:water (0.1:5:4.9, *v*/*v*).

### 2.5. Chemical Analysis and Monosaccharide Composition

Total sugars were estimated using the phenol-H_2_SO_4_ reaction [22] with L-fucose as the standard. After acid hydrolysis of polysaccharides (4 M HCl, 100 °C, 6 h), the sulfate content was determined according to the gelatin-barium method [23], using sodium sulfate as a standard. Protein content was measured using Spector’s method [24]. To determine the best polysaccharide acid hydrolysis conditions when using HCl, that is, where polymer degradation occurs without destroying the monosaccharides released by this degradation, polysaccharides from *S. schröederi* were hydrolyzed with HCl at concentrations of 0.5 M, 1 M, 2 M, and 4 M, for 30 min, 1 h, 2 h, and 4 h, respectively. A temperature of 100 °C was maintained for all hydrolysis conditions. The material was later neutralized, dried, and resuspended in water, and reducing sugars were determined using the Somogyi–Nelson method [25]. In all cases, the best hydrolysis condition was 2 M HCl for 2 h at 100 °C. Thus, polymers were hydrolyzed using those conditions and their sugar composition was determined using a LaChrom Elite^®^ HPLC system (Hitachi Co., Tokyo, Japan) with a refractive index detector (RI detector model L-2490). A LichroCART^®^ 250-4 column (250 mm × 40 mm) packed with Lichrospher^®^ 100 NH2 (5 µm) was coupled to the system (Both columns are from Merck Co., Darmstadt, Germany). The sample mass used was 0.2 mg and the analysis time was 25 min. The following sugars were analyzed as a reference: arabinose, fructose, fucose, galactose, glucose, glucosamine, glucuronic acid, mannose, mannuronic acid, rhamnose, and xylose.

### 2.6. Nanoparticle Synthesis

Nanoparticles were synthesized according to a previously described method [16,17]. F0.5v (the fraction that contains fucan A) was used as a bioreductor in this experiment. Briefly, a solution of F0.5v (10 mg/mL) was added to a silver nitrate solution (1 mM in ultrapure water) at a proportion of 1:9 and left to rest. The solution was subjected to continuous stirring for 1 h and was protected from light. Afterwards, the mixture was centrifuged at 10,000× *g* for 15 min at 4 °C. The precipitate was collected and suspended in ultrapure water and lyophilized. The precipitate obtained was composed of silver nanoparticles containing fucan A (AgFuc). To determine the yield of the AgFuc synthesis method, the amount of F0.5v used was considered to be 100%.

### 2.7. Characterization of the Nanoparticles

#### 2.7.1. UV-Visible Spectroscopy

The formation of AgFuc nanoparticles and the reduction of silver were analyzed by observing the change in color of the solution, from light yellow to dark brown. Electron spectroscopy in the UV-visible region with readings in the 350 to 800 nm range was performed to monitor these reactions using the UV/Visible spectrophotometer DR 5000 Hach Lange GmbH (Düsseldorf, Germany).

#### 2.7.2. Fourier-Transform Infrared Spectroscopy (FT-IR)

The samples (10 mg) were mixed with KBr and triturated. Next, the obtained pellets were subjected to FTIR. The sample spectra were obtained using a spectrophotometer (IRAAffinity-1 Shimadzu Corp., Kyoto, Japan) equipped with the IRsolution Shimadzu Corp. software (Kyoto, Japan) version 1.60 using a scan number of 32 and a resolution of 4 cm^−1^. The frequency range for the analysis was 4000–400 cm^−1^. The analyses were carried out at the analytical center of UFRN’s Chemistry Institute and the Coordination and Polymers Chemistry Laboratory (Laboratório de Química de Coordenação e Polímeros—LQCPol—Department of Chemistry) of the Federal University of Rio Grande do Norte (Universidade Federal do Rio Grande do Norte—UFRN). Three independent analyses were performed.

#### 2.7.3. Atomic Force Microscopy (AFM), X-Ray Diffraction (XRD), Dynamic Light Scattering (DLS), and Scanning Electron Microscopy (SEM) Analysis

AFM was performed using a scanning probe microscope (SPM) 9700 (Shimadzu, Kyoto, Japan) at the Scanning Electron Microscopy Laboratory (LABMEV) of the Department of Engineering Materials (DEMat) of UFRN. At least three images were taken from different fields. All images were evaluated for the shape of the nanoparticles in the samples. One drop of the AgFuc suspension (0.5 mg/mL) was dried on a glass coverslip and subjected to AFM analysis.

XRD analysis was used to assess the behavior of the crystallographic phases to verify whether the nanoparticle had a characteristic crystalline plane. An XRD 7000 X-ray diffractometer (Shimadzu, Kyoto, Japan) was used, with CuKα radiation equal to 1.5406 Å, a 2Ɵ diffraction angle in the range of 20–80°, an angular speed of 5°/min, and an interval of 0.02°.

The AgFuc nanoparticles were analyzed using DLS. The hydrodynamic diameter, polydispersity index (PDI), and zeta potential were determined using a zeta potential analyzer (Brookhaven, New York, NY, USA). Briefly, AgFuc suspensions (0.5 mg/mL) were analyzed in three independent experiments; the reported values correspond to the mean ± standard deviation (SD).

AgFuc nanoparticles were processed and analyzed via SEM (Shimadzu SSX550; Shimadzu Corp. Kyoto, Japan). Briefly, a 20 μL volume of AgFuc (0.5 mg/mL) nanoparticles was loaded on a carbon-coated copper grid without gold coating and air-dried for 10 min under a vacuum. The grid chamber was then placed in the SEM room and incubated in the dark at 10–20 °C for 2 h. Finally, the grid chamber was loaded on the SEM stage for analysis and images were captured from different zones with different resolutions. Representative images of three independent experiments are shown.

#### 2.7.4. Quantitative Determination of Silver in AgFuc Nanoparticles by ICP-OES

##### Digestion of AgFuc Nanoparticles

Digestion was carried out in a microwave digester (Milestone S.r.l.—START D from, Shimadzu Co., Tokyo, Japan) as described in Amorim et al. [16]. Briefly, nitric acid (67%) was purified by sub-boiling distillation (Berghof, Eningen, Germany). Afterwards, 7 mL of this acid and 1 mL of 30% H_2_O_2_ (Merck, Darmstadt, Germany) were dropped into a teflon cup containing the sample (100 mg). The cup was closed and submitted to digestion as described in [16]. Following this process, the digested material was suspended in deionized water (15 mL). Insoluble particles were removed after filtration (0.45 μm). The experiments were conducted in duplicate and analytical blanks were obtained by conducting the procedure in the absence of a sample.

##### Determination of the Silver Content of the Digested AgFuc Nanoparticles

An inductively coupled plasma emission spectrometer (ICP-OES 5100 VDV, Agilent Technologies, Tokyo, Japan) was used to quantify the silver content of the digested AgFuc as described in Amorim et al. [16]. Argon with a purity of 99.996% (White Martins, São Paulo-SP, Brazil) was used as the feed gas, with a gas flow of 12.0 L/min and 1.0 L/min, principal and auxiliary argon flow, respectively. For the ICP-OES equipment, the parameters used during the analysis were a plasma power of 1.5 kW, nebulization flow 0.70 L/min, stabilization and reading time 15 s, and wavelength of 328,068 nm; three replications were carried out. A reference standard solution of silver (Merck, Darmstadt, Germany) was made using 5% (*v*/*v*) nitric acid prepared from 65% acid distilled in a sub-boiling system (Distillacid, Berghof, Eningen, Germany). Several silver solutions were obtained from this standard solution, ranging from 2.5 to 100 g/L, and were used to create the analytical curve (*r* = 0.9999). Serial dilutions of 0.1 mL/10 mL and 0.2 mL/10 mL were prepared for sample measurement.

### 2.8. Evaluation of the Antiparasitic Activity of AgFuc Nanoparticles

The epimastigote forms of the Y strain of *T. cruzi* were maintained in LIT media supplemented with 10% FBS at 28 ± 2 °C in a biochemical oxygen demand (BOD) kiln. The strains were cultured until the exponential phase of growth (1 × 10^7^ parasites/mL) was reached. AgFuc nanoparticles and F0.5v were tested at concentrations of 100, 50, 25 µg/mL. In addition, Ag (silver) was evaluated. However, as AgFuc is made up of 4.8% silver, Ag was evaluated at lower concentrations (5, 2.5, 1.0, 0.5, 0.25, and 0.1 µg/mL) than AgFuc. Therefore, 90 μL of the culture (1 × 10^7^ parasites) and 10 μL of the sample suspension or solution were added to a 96-well plate and incubated at 28 ± 2 °C for 24 and 48 h. After incubation, 20 μL of resazurin (1 mM) was added and incubated for 24 h in a BOD kiln. Subsequently, the absorbance was read at 570 and 600 nm using an Epoch Microplate Spectrophotometer obtained from BioTek (Winooski, VT, USA). The parasites exposed to the test conditions in the absence of treatment were used as a control. The assays were performed in triplicate, and the data obtained were processed using GraphPad Prism software version 5.0, 2014 (La Jolla, CA, USA). The inhibition rate was determined using the following formula:

% inhibition = 100 − [𝐴570t − (𝐴600𝑡 × 𝑅0)/𝐴570c − (𝐴600𝑐 × 𝑅0)] ∗ 100(1)

where *A*570t is the sample optical density (OD) at 570 nm, *A*600t is the sample OD at 600 nm, *A*570c is the control OD at 570 nm, *A*600c is the control OD at 600 nm, and RO is the factor used to eliminate the influence of the medium on the readings, obtained using the following formula:

𝑅0 = 𝐶𝑚edium 570 nm/𝐶𝑚edium 600 nm(2)

where *Cm*edium 570 nm is the absorbance of the medium at 570 nm and *Cm*edium 600 *nm* is the absorbance of the medium at 600 nm.

### 2.9. Flow Cytometry

Whether the mechanism of *T. cruzi* cell death involved apoptosis or necrosis was investigated via labeling with Annexin V-FITC/PI using an Annexin V FITC Apoptosis Detection Kit (Invitrogen) according to the manufacturer’s instructions. Epimastigote forms of the *T. cruzi* Y strain (1 × 10^7^ cells/mL) were cultured in LIT media supplemented with 10% FBS and treated with AgFuc nanoparticles (50 µg/mL) for 24 h. The parasites were then centrifuged at 2000 rpm at 4 °C for 5 min. The obtained pellet was then washed with 200 μL phosphate-buffered saline (PBS), resuspended in 100 μL binding buffer, and labeled with Annexin V/PI for 10 min at 25 °C in the dark. The analysis was performed on a FACSCanto II flow cytometer (BD Biosciences, Eugene, OR, USA) with FACSDiva software, version 6.1.2 (Becton Dickson, Franklin Lakes, NJ, USA). For each experimental condition, 20,000 events were captured; the percentage of labeled cells was calculated using FlowJo Software, version vX.0.7 1997–2014 (FlowJo, Ashland, OR, USA). Cells not exposed to sample were used as control.

### 2.10. Analysis of Mitochondrial Damage in Parasites Treated with AgFuc Nanoparticles or F0.5v

To analyze the mitochondrial damage to the parasite in response to treatment with AgFuc and F0.5v, the mitochondrial potential marker rhodamine 123 (CAS Number: 62669-70-9) was used according to the manufacturer’s guidelines (Sigma Chemical Company, St. Louis, MO, USA) with modifications. The parasites were centrifuged at 2000 rpm for 6 min at 4 °C and washed with ice-cold PBS. Subsequently, they were suspended in 200 µL of PBS and 0.5 µL of rhodamine (5 mg/mL). After 15 min, the parasites were evaluated by flow cytometry. The analysis was performed on a FACSCanto II flow cytometer (BD Biosciences, Eugene, OR, USA) with the FACSDiva software, version 6.1.2 (Becton Dickson, Franklin Lakes, NJ, USA). For each experimental condition, 10,000 events were captured, and the percentage of labeled cells was calculated using the FlowJo Software Version vX.0.7 1997–2014 (FlowJo, Ashland, OR, USA).

### 2.11. Statistical Analysis

All data from the experiments are expressed as mean ± standard deviation (*n* = 3) and results are from three independent assays. The data are representative of three separate tests carried out independently. Statistical analysis was performed using GraphPad Prism 5 software (GraphPad Prism, version 5.00, San Diego CA, USA). Results are expressed as the mean ± SD. Data analysis was performed using one-way analysis of variance (ANOVA) followed by Tukey’s test. Data are expressed as mean ± standard deviation, and a *p*-value lower than 0.05 (*p* < 0.05) was considered significant.

## 3. Results and Discussion

### 3.1. Obtaining Fucan A

The seaweed *S. schröederi* synthesizes three populations of fucoidans that have been named according to their mobility in agarose gel electrophoresis, namely, fucan A, fucan B, and fucan C [20]. Fucan A accounts for ~80% of the fucoidans synthesized by this seaweed [26]. In addition, this fucoidan does not show genotoxicity or mutagenicity in either in vitro [27] or in in vivo animal studies [28]. As such, fucan A needs to be separated from the other two types of fucans. For this purpose, the extract was subjected to sequential fractionation using propanone. Fucan A can be separated using low volumes of propanone, and is found mainly in the first two fractions, called F0.5v and F0.6v [16,20,27,28,29].

Therefore, using the methodology described above, *S. schröederi* fucans were extracted and fractionated by differential precipitation with propanone; we obtained seven fractions that were rich in fucoidans. The yield (%) and denomination of the fractions used in this work were as follows: F0.5v (43.5% ± 0.22), F0.6v (11.8% ± 0.16), F0.7v (5.9% ± 0.04), F0.9v (8.0% ± 0.03), F1.1v (4.29% ± 0.1), F1.3v (0.65% ± 0.01), and F2.0v (4.46% ± 0.03).

### 3.2. Chemical Composition of Samples

The chemical composition of these fractions is shown in Table 1. The presence of fucose and sulfate in all samples indicates that fucoidans were present in all seven fractions.

**Figure 1:**
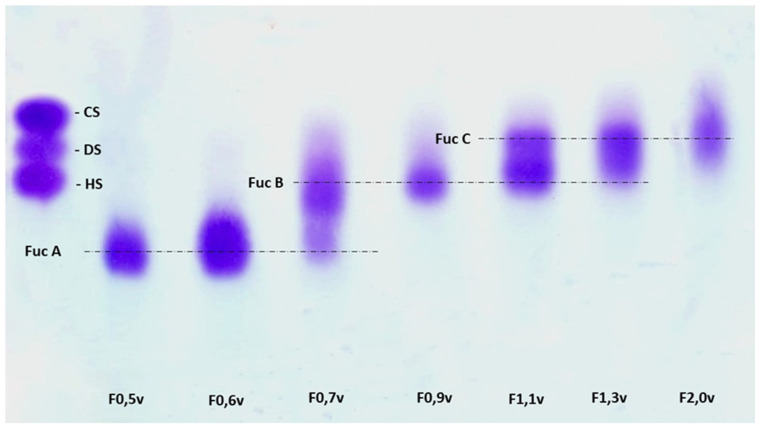
Electrophoresis in 0.05 M diaminopropane acetate buffer, pH 9.0 of fucoidans obtained by propanone precipitation. Approximately 5 µL (50 µg) of each polysaccharide was loaded onto an agarose gel prepared in diaminopropane acetate buffer and subjected to electrophoresis. Fuc A—fucan A; Fuc B—fucan B; Fuc C—fucan C; CS—chondroitin sulfate; DS—dermatan sulfate; HS—heparan sulfate. This figure is representative of three separate tests made independently.

The obtained fractions were then subjected to agarose gel electrophoresis and later stained with toluidine blue. The electrophoretic profiles of the fractions are shown in Figure 1, which shows that, as previously described [16,20,27,28], fucan A is mainly found in F0.5v and F0.6v. Although Fucan A is present in F0.7v, this fraction is contaminated with fucan B. Fucan B acts as a contaminant for fucan C as well (F1.1v and F1.3v); however, F0.9v contains only fucan B, while in the case of fucan C, it can be isolated from F2.0v, which is not contaminated with other fucoidan types.

Generally, in agarose gel electrophoresis, the mobility of the polysaccharides depends on their charge; for example, a higher negative charge leads to greater mobility. However, as suggested by Dietrich and Dietrich [30], the use of 1,3-diaminopropane is important for the visualization of the different polysaccharide molecules present in the fractions. At the pH at which the experiment was conducted (9.0), sulfated polysaccharides, including fucoidans, assume a conformation in which certain sulfate groups are exposed and others are not. These exposed sulfates react with the diamine and their negative charges are quenched. This induces a change in the conformation of the polysaccharides, leading to the exposure of new sulfate groups that react with the diamine in the buffer. This process continues until equilibrium is reached and the conformation of the polysaccharides no longer changes. However, polysaccharides that reach this conformational equilibrium have sulfate groups that did not react with the diamine, and it is these groups that influence the mobility of the polysaccharide during agarose gel electrophoresis. In short, polysaccharide molecules with the same structure have the same interactions with diamine, and consequently have the same electrophoretic motility, whereas structurally different polysaccharides have different electrophoretic mobilities [30]. Therefore, we were able to separate three sulfated glycosaminoglycans, namely, heparan, dermatan, and chondroitin (Figure 1), which, while structurally similar, have different conformations. Therefore, the fact that there was only one band in the F0.5v and F0.6v fractions indicates that these fucoidans were isolated from other seaweed fucoidans and that these samples were made up of the same polysaccharide, in this case, fucan A.

### 3.3. Synthesis of Silver Nanoparticle Containing Fucan A (AgFuc)

**Figure 2:**
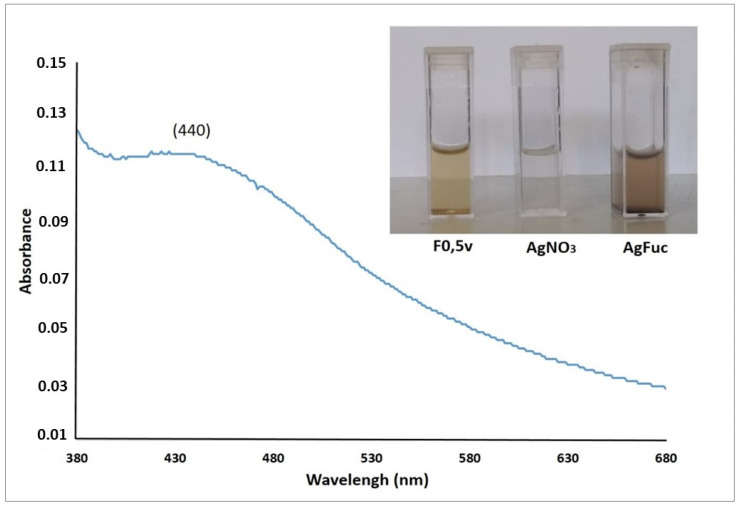
UV–vis absorption spectrum of the AgFuc suspension. F0.5v suspension (F0.5v), silver nitrate 1 mM solution (AgNO_3_), and AgFuc suspension (10 mg/mL). The darker color in the AgFuc suspension is related to the reduction of the silver particles. These figures are representative of three separate tests made independently.

The small square in Figure 2 shows an image of the F0.5v fraction and the silver nitrate solutions. After the two were combined and the nanoparticle formation process was complete, a darkening of the solution was observed, confirming the formation of nanoparticles.

The light absorption profile of AgFuc was evaluated by UV-vis spectroscopy using wavelengths between 300 and 700 nm (Figure 2). An increase in absorption was observed in the region between 430 and 450 nm. From this region, a decrease in the absorbance was observed that almost reached zero during the sweep. When the silver nitrate or fucoidan solutions were analyzed separately in the 400–450 nm range, the same light absorption profile was not observed in this wavelength range (data not shown). This increase indicates the formation of silver nanoparticles, as the increase in absorbance is due to surface plasmon resonance, which is a common event in metallic nanoparticles [31]. Moreover, in the case of nanoparticles, an increase in absorption in this region of the spectrum indicates that the nanoparticles have a rounded shape [32].

### 3.4. Characterization of AgFuc

**Figure 3:**
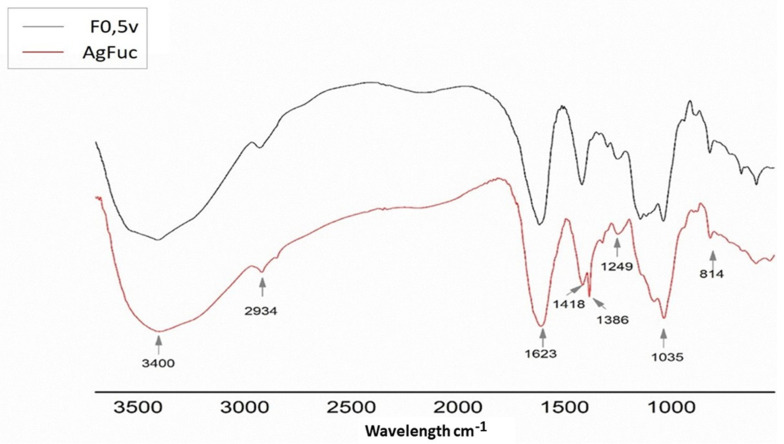
Overlapping infrared spectra for F0.5v (in black) and AgFuc (in red) between 4000 and 400 cm^−1^. This figure is representative of three separate tests made independently.

The infrared spectra for the F0.5v fraction and AgFuc are shown in Figure 3. The bands observed at 3400 cm^−1^ can be attributed to the O-H stretch present in the samples. It is important to emphasize that a free O–H stretch appears at higher frequencies because greater energy is required to create vibrations. Sp3 C-H stretches were confirmed, with bands at 2934 cm^−1^. The band at approximately 1623 cm^−1^ can be attributed to the carbonyl groups of the monosaccharides, which overlap with the H_2_O deformation signals. The presence of sulfate in both samples is confirmed by the bands at 1249, 1035, and 814 cm^−1^, which correspond to the vibrations of asymmetric S = O stretching, symmetric C–O associated with a C–O–SO_3_ group, and C-O-S of the sulfate groups at the axial position, respectively. The bands observed in the 1463–1220 cm^−1^ region can be attributed to the angular deformation of the CH groups and the stretching vibration of CO, respectively. These bands have previously been described in the FTIR spectra of fucan A [26,28], confirming the presence of this fucan in the AgFuc nanoparticles.

In addition to the similar bands found in the spectra of the two samples, it was observed that the AgFuc spectrum contained a band at 1386 cm^−1^ that corresponds to reduced silver [33]. Bands in this region have been described in other silver nanoparticles that contain fucans isolated from brown seaweed [34].

**Figure 4:**
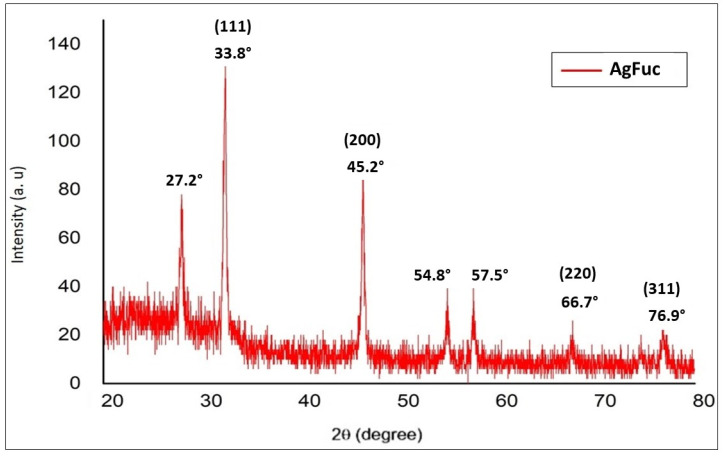
XRD patterns of AgFuc nanoparticles. This figure is representative of three separate tests made independently.

The X-ray diffraction (XRD) patterns of the obtained AgFuc particles are shown in Figure 4. The diffraction peaks at 33.8°, 45.2°, 66.7°, and 76.9° have a strong intensity and resemble the planes of silver crystals (111), (200), (220), and (311), respectively, according to JCPDS no. 04-0783 [35].

For 10 mg of fucan A, 4 mg of AgFuc were obtained. The constituents of the AgFuc nanoparticles were determined using an inductively coupled plasma emission spectrometer as described in the Methods section, and the particles were found to consist of sugar (~95%) and silver (~4.8%). Several previous authors have verified the amount of silver in the structure of nanoparticles containing polysaccharides. In studies conducted by Amorim et al. [16], 6.7% silver was found in the composition of a fucan-coated nanoparticle. Silver nanoparticles containing xylan synthesized using the same method employed in this study have been shown to contain 19% silver and 81% polysaccharides (xylan) [36]. This indicates that the efficiency of silver nanoparticles synthesis as well as the characteristics of the synthesized nanoparticles depend on certain factors, such as temperature, the concentration of the reducing agent used during the process, and the reaction time [37], which explains both the previously-described variation in values and the variation presented in this study.

**Figure 5:**
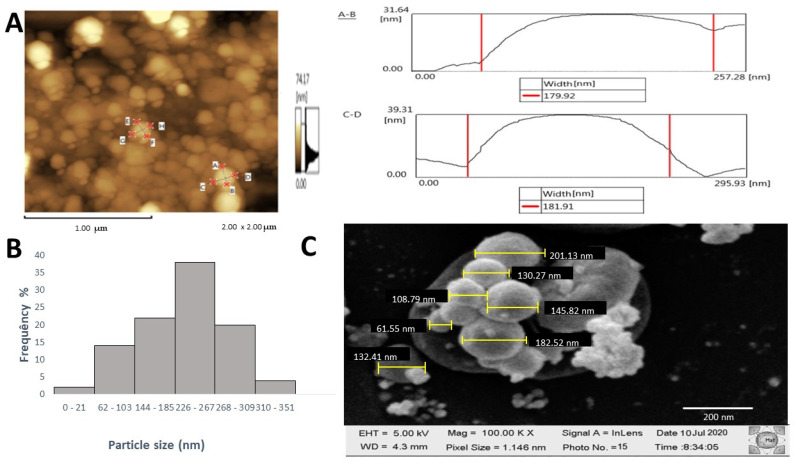
Physical properties of AgFuc particles: (**A**) AFM images of AgFuc particles; (**B**) size dispersion histogram obtained using dynamic light scattering (DLS); (**C**) SEM images of the AgFuc particles. A-B and E-F nanoparticle width; C-D and G-H nanoparticle length. These figures are representative of three separate tests made independently.

Using atomic force microscopy (AFM), we were able to verify the rounded shape of the AgFuc nanoparticles as well as their diameter, which was approximately 180 nm (Figure 5A). The mean hydrodynamic diameter of AgFuc nanoparticles in water as measured by dynamic light scattering (DLS) was 283 nm. However, we identified particles with sizes ranging from 30 to 350 nm (Figure 5B). Figure 5C shows a scanning electron microscopy (SEM) image of AgFuc nanoparticles. The particles were predominantly spherical in shape, and had an average size of 155.3 ± 10 nm. The size of the AgFuc nanoparticles, as determined by AFM/SEM, is smaller than that found using DLS measurements, which was expected as DLS measures the hydrodynamic radius of the nanoparticles suspended in water as well as any coating material on the surface of the nanoparticle [38]. In addition, these data corroborate the findings of Venkatesan et al. [39], who authors synthesized a chitosan–fucoidan complex-coated silver nanoparticle using a green method. They observed that this nanoparticle appeared to differ in size according to the method of analysis used. As in the present study, their chitosan–fucoidan complex-coated silver nanoparticle was found to be smaller with SEM than with DLS tests due to the chitosan–fucoidan capping agent. The difference in size obtained here between the AFM/SEM and DLS data can therefore be attributed to the capping agent present on the surface of AgFuc nanoparticles, namely, fucan A.

Zeta potential analysis revealed that AgFuc has a surface charge of −27.4 ± 1.8 mV. Negative values for the zeta potential have already been described in silver nanoparticles containing fucoidans, attributed to the presence of negative charges from the sulphate groups present in fucoidans [17,39].

The cytotoxicity of silver nanoparticles is influenced by variations in particle size. Studies report that smaller particles can induce higher cytotoxicity because they can be easily internalized by cells. In support of this hypothesis, smaller nanoparticles (15 nm) have been shown to generate more reactive oxygen species in murine macrophage lines than silver nanoparticles with sizes of 30 and 55 nm [40]. In addition, silver nanoparticles with a diameter of 5 nm were more toxic than those 20 and 50 nm in diameter in several human cell lines [41]. Therefore, the size of AgFuc nanoparticles is in the expected range for particles that are less cytotoxic.

Morphology is another factor that influences the activity of different types of silver nanoparticles. The most commonly obtained structures are spherical, triangular, square, cubic, rectangular, oval, and acicular. In terms of their toxicity, it is not yet known which particle morphologies have the best effect on biological systems, as this depends on several factors such as concentration, pH, temperature, and electric charge. However, studies have already observed that spherical silver nanoparticles such as the AgFuc nanoparticle studied in this work do not have damaging effects on cells [42]. In addition, most silver nanoparticles containing fucans and fucoidans have been described as being spherical in shape [43].

To verify the stability of the AgFuc particles, their size was periodically measured over a five-month period. The results are shown in Table 2. The mean size of AgFuc nanoparticles was 283.8 ± 15.2 nm at month one and 245.1 ± 14.72 nm at month five. In addition, the polydispersity index (PDI) ranged from 0.32 to 0.35 during this time. This suggests that the AgFuc nanoparticles were reasonably stable. One of the factors that contributes to the stability of nanoparticles is surface charge. Nanoparticles that assume positive or negative charges show resonance, which prevents their aggregation, thereby keeping both their sizes and their stability in suspension constant [44]. In this way, the surface load (zeta potential) of the AgFuc nanoparticles was determined; we found an average value of −7.9 ± 1.25, showing good stability over a five-month period.

### 3.5. Evaluation of the Antiparasitic Activity of AgFuc

The antiparasitic activity of Ag (silver), the F0.5v fraction, and AgFuc nanoparticles against the *T. cruzi* Y strain is shown in Table 3. The tested compounds were ineffective at the smallest concentration evaluated (25 µg/mL). Moreover, among all of them, silver had the lowest cytotoxicity against the parasite, inhibiting only approximately 25% of the parasites’ ability to reduce resazurin. F0.5v only affected the parasites at the highest concentration tested (100 µg/mL). In addition, its effect was greater after 48 h of exposure. AgFuc nanoparticles had the highest cytotoxic effect at the two highest concentrations tested, and this effect was shown to be time-dependent.

Another interesting finding is that F0.5v in the form of nanoparticles was shown to have enhanced activity. Thus, we compared its effects with those of AgFuc nanoparticles at the same concentration of 50 µg and after 48 h of exposure. However, it must be kept in mind that 50 µg of AgFuc nanoparticles contain approximately 45 µg of F0.5v and 5 µg of silver, as determined by inductively coupled plasma emission spectroscopy (ICP-OES). When comparing the activity of AgFuc and F0.5v nanoparticles, AgFuc appears to have an inhibitory action that is ten times greater than that of F0.5v. Moreover, in the case of silver, AgFuc has an inhibitory effect which is four times greater, although the comparison was made using silver at concentrations which were ten times higher than those found in the AgFuc nanoparticles. In addition, in all evaluated conditions AgFuc was more efficient than BNZ, a drug used in the treatment of Chagas disease; in addition, it was more efficient than a mix of Ag and F0.5v even though it contains the same amount of compounds present in AgFuc. This indicates that fucoidan and silver are much more effective as anti-*T. cruzi* in nanoparticle form than in the form of isolated molecules.

This difference between the effects of the samples was less pronounced when a higher concentration (100 µg/mL) was used. Therefore, the following tests were performed with 50 µg/mL.

### 3.6. Evaluation of the Cell Death Process via Annexin V-FITC and Propidium Iodide (PI) Labeling

**Figure 6:**
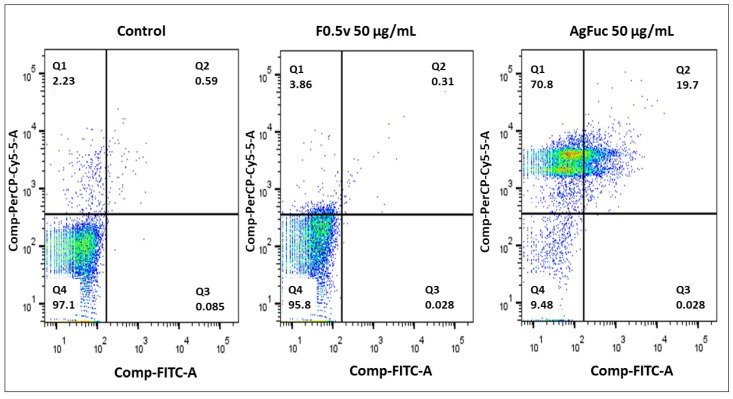
Evaluation of the process of cell death through flow cytometry using untreated parasites and parasites treated with AgFuc or F0.5v for 24 h. After the treatment period, the *T. cruzi* parasites were labeled with Annexin V-FITC and propidium iodide (PI) and analyzed using flow cytometry. Control corresponds to cells exposed to medium without any sample. These figures are representative of three separate tests made independently.

In order to examine whether the data observed in the resazurin test were correlated with cell death, an Annexin V-FITC and PI labeling assay was performed; this assay can help to differentiate apoptotic, necrotic, and viable cells according to the labeling pattern. Y Strain *T. cruzi* were treated with AgFuc or F0.5v at concentrations of 50 μg/mL and analyzed by flow cytometry. As shown in Figure 6, in the control group, 97.1% of cells were negative for Annexin V-FITC and PI labeling.

F0.5v (50 μg/mL) did not induce parasite death, confirming the data obtained in the resazurin test and demonstrating that the parasites are resistant to the action of F0.5v. On the other hand, the presence of AgFuc greatly increased the number of cells stained with PI (indicative of necrosis) compared to the control group. In addition, a high number of cells marked with annexin (19.7%) were observed, which is indicative of apoptosis.

We identified only one other paper that evaluated silver nanoparticles containing polysaccharides as an anti-Trypanosome agent. Brito et al. [36] showed that treatment of *T. cruzi* with silver nanoparticles containing xylan (xylose-rich polysaccharide) resulted in 98% of cells being labelled with PI. However, the authors of this study were unable to suggest a mechanism of action for the nanoparticles used.

### 3.7. Analysis of the Damage in the Parasites’ Mitochondria after Treatment with F0.5v or AgFuc

**Figure 7:**
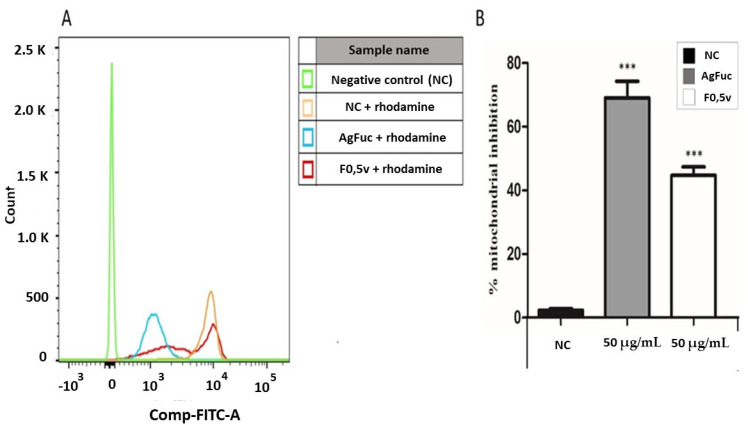
Analysis of mitochondrial damage caused by AgFuc or F0.5v in the *T. cruzi* after 24 h of treatment. (**A**)—The parasites were exposed to samples for 24 h, after they were suspended in 200 µL of PBS and 0.5 µL of rhodamine (5 mg/mL). After 15 min, the parasites were evaluated by flow cytometry. (**B**)—The percentage of labeled parasites was quantified, and the graph shows the mean ± standard deviation of the percentage of labeled inhibition. *p* < 0.001 (***) control vs. samples. NC - Negative control corresponds to parasites exposed to PBS without rhodamine. These figures are representative of three separate tests made independently.

Figure 7A shows a histogram of the parasites stained with rhodamine 123, which is a marker of mitochondrial function. Moreover, Figure 7B shows the percentage of mitochondrial inhibition as measured by labelling with rhodamine after treatment with AgFuc or F0.5v compared to the untreated controls. We found that F0.5v treatment led to a 40% reduction in mitochondrial staining. However, the presence of AgFuc led to an 80% inhibition of mitochondrial labeling. This indicates that AgFuc induces significant mitochondrial damage, which can explain the large number of dead cells (Figure 6) observed after treatment with AgFuc nanoparticles.

*T. cruzi* and other microorganisms form a group of single-cell flagellate eukaryotes named kinetoplastids. Their main distinguishing feature is the presence of a kinetoplast, a DNA-containing granule inside the single mitochondrion at the base of the cell’s flagella (the basal body) [45]. This characteristic makes the kinetoplast a target for the development of anti-*T. cruzi* drugs [46].

To the best of our knowledge, no other study has suggested a possible mechanism that underlies the anti-trypanosome activity of silver nanoparticles. We believe that the parasite may have receptors that recognize several polysaccharides, such as fucoidans, and that this promotes the entry of the nanoparticle into the parasite’s cytoplasm. In this environment, the nanoparticles decompose; in much the same way as in bacteria, the silver component induces the formation of reactive species that lead to mitochondrial (kinetoplast) damage, and the consequent death of the parasite. On the whole, the data shown in Figure 7 provide insights into the mechanisms of action of AgFuc. However, additional studies are required in order to fully understand this mechanism. In addition, there are several issues, which need to be addressed, such as the exact mechanism of interaction of silver nanoparticles with the parasite cells, how the surface area of the nanoparticles influences their killing activity, and more.

In addition, the efficiency of antibacterial silver nanoparticle activity can be increased by lowering the particle size. As seen from the AgFuc characterization, particularly UV-vis analysis and the size-dispersion histogram, the particles are rather polydisperse. Therefore, the larger AgFuc nanoparticles may not enter the cytoplasm of the parasites, and this may negatively affect the anti-trypanosome action of AgFuc. In future studies, we intend to determine the best conditions to obtain AgFuc nanoparticles with a smaller size and to evaluate the effect this has on the survival of *T. cruzi*.

In our previous paper, we showed that similar AgFuc nanoparticles did not show any toxic effects against various non-tumor cell lines while decreasing the viability of various tumor cells, especially renal adenocarcinoma 786-0 cells. In addition, assays performed with several non-tumor kidney cell lines (HEK, VERO, MDCK) showed that these nanoparticles only induce death in tumor renal 786-0 cells [16]. Regarding *S. schröederi* fucoidan (Fuc A), in a previous paper it was demonstrated that this polysaccharide (from 20 to 2000 µg/g body weight per rat for seven days) did not cause general adverse effects and mortality. Regarding subchronic study, fucan A did not cause any change in hematological and biochemistry parameters, morphology, or size of rats’ organs analyzed at a concentration of 20 µg/g body weight per rat during a 62-day period [27]. In addition, Fuc A showed no mutagenic activity in a Salmonella reversion assay when the bacterial strains TA97a, TA98, TA100 and TA102 (with and without S9) were used. A comet assay showed that Fuc A had no genotoxic effect (from 20 to 1000 mg/mL) on CHO cells [28], indicating that Fuc A is a safe compound that can be used for the development of silver nanoparticles with anti-*T. cruzi* action.

In another study, male CD-1(ICR) mice were treated with AgNPs of different sizes (10 nm, 40 nm, 100 nm), citrate-or polyvinylpyrrolidone-coated. The authors showed that the administration of the smallest (10 nm) nanoparticles resulted in enhanced silver tissue distribution and overt hepatobiliary toxicity compared to larger ones (40 and 100 nm), while coating had no relevant impact [47]. Therefore, we believe that AgFuc can reach multiple organs and be less toxic than silver nanoparticles that are smaller in size.

To date, the effects of AgFuc have not been evaluated. However, based on data from the literature, it is possible to suggest events related to the pharmacokinetics of these nanoparticles. It is likely that if they are administered orally, they can be absorbed via paracellular transport, transcytosis, and M cell uptake in the gastrointestinal tract, as opposed to mainly subcutaneous, intramuscular, or inhaled nanoparticles, which are absorbed via macrophages and lymphatic uptake [47,48]. The liver has been described as the primary organ for AgNP distribution, followed by the spleen and kidneys, regardless of whether exposure is oral, intravenous, subcutaneous, or through inhalation [49]. However, AgNPs can be transported to different organs via blood circulation and distributed in many systems, such as the dermis, respiratory system, spleen, digestive system, immune system, and muscular (skeletal, smooth, and cardiac muscles) system [49].

This biodistribution of AgNPs is important in the case of combating *T. cruzi*, as this parasite infects cells from many different tissues. We hope in the future to carry out an in vivo study to obtain further data about AgFuc, including its pharmacokinetics.

Regarding the metabolization of silver from AgFuc, Ag+ can react with reduced glutathione (GSH) to produce Ag–GSH polymer complexes, followed by partitioning to various tissues. In addition, AgNPs can be sulphidated to produce Ag_2_S NPs, which can interact with selenium to produce Ag_2_Se NPs and Ag/S/Se argyrial particulate [47]. Following oral administration, the faecal excretion rate has been reported to be 63% and 49% for 12 nm nanoparticles and ionic silver, respectively [50]. Recordati et al. [51] reported urinary excretion of silver at 12 h of an orally administered of silver nanoparticles. Although there are no in vivo studies involving AgFuc, we expect AgFuc silver metabolization and excretion to occur in a similar way; however, future studies are required to confirm this assumption.

## 4. Conclusions

The identity of a fucoidan called Fucan A, present in sample F0.5v, was confirmed by different chemical analyses, agarose gel electrophoresis, and infrared spectroscopy. Moreover, using F0.5v we were able to synthesize silver nanoparticles using an environmentally friendly method. These nanoparticles (AgFuc) have a rounded shape, an average size of 180 nm, and consist of silver (~5%) and fucan A (~95%). AgFuc nanoparticles were shown to be more toxic to *T. cruzi* than silver or F0.5v alone. In addition, AgFuc nanoparticles promoted alteration of the mitochondrial function of *T. cruzi*, leading to death via necrosis. The kinetoplast of *T. cruzi* represents an important pharmacological target for the development of antiparasitic drugs; as such, our data represent a starting point in the potentiation of AgFuc nanoparticles for the development of new drugs to treat Chagas disease.

## Figures and Tables

**Table 1:** Chemical composition of fucoidans from *S. schröederi*.

Sample	Sugar (%)	Proteins (%)	Phenolic c. (%)	Molar Ratio ^a^					
				Fuc	Xyl	GluA	Gal	Man	Sulfate
F0.5v	88.2 ± 1.9	0.03 ± 0.04	0.01 ± 0.01	1.0	0.5	4.0	nd	nd	1.3
F0.6v	87.1± 0.9	0.04 ± 0.01	<0.01	1.0	0.6	4.0	0.3	0.2	1.3
F0.7v	85.2 ± 2.3	nd	<0.01	1.0	0.9	0.5	1.3	0.1	1.7
F0.9v	79.3 ± 3.9	nd	<0.01	1.0	0.6	0.2	1.6	nd	2.0
F1.1v	78.7 ± 0.9	nd	<0.01	1.0	0.7	0.1	1.3	nd	2.0
F1.3v	77.6 ± 1.7	nd	nd	1.0	1.1	nd	0.7	nd	2.2
F2.0v	77.0 ± 5.9	0.03 ± 0.01	nd	1.0	0.4	nd	1.0	nd	2.3

nd: not detected; Xyl: xylose; Fuc: fucose; Gal: galactose; Man: mannose; GlucA: glucuronic acid. ^a^ Analyzed by HPLC after acid hydrolysis at 100 °C for 2 h. Phenolic C.: phenolic compounds.

**Table 2:** Average size of the AgFuc particles evaluated over a five-month period.

Month	AgFuc Diameter (nm)	Polydispersity Index
1	283.8 ± 15.20	0.32 ± 0.03
2	278.5 ± 21.02	0.33 ± 0.02
3	267.9 ± 19.72	0.30 ± 0.04
4	261.4 ± 15.41 *	0.31 ± 0.02
5	245.1 ± 14.72 *	0.35 ± 0.03

*p* < 0.01 (*) compared to the control group.

**Table 3:** Evaluation of the antiparasitic activity of Ag, F0.5v, and AgFuc nanoparticles against the epimastigote evolutionary form of the *T. cruzi* Y strain.

	% Inhibition			
	Samples	25 µg/mL	50 µg/mL	100 µg/mL
24 h	BNZ	6.1± 1.3 *^a^	20 ± 2.1 **^a^	23 ± 1.3 **^a^
	F0.5v	0.0	0.0	43.0 ± 1.1 ***^b^
	AgFuc	0.0	14.1 ± 2.2 **^a^	58.9 ± 1.2 ***^c^
	Ag	4.4 ± 1.3 *^a^	10.0 ± 1.7 **^a^	14.4 ± 0.85 **^a^
	F0.5v:Ag (9:1)	0.0	0.0	40.0 ± 0.8 ***^b^
	Control #	0.0	0.0	0.0
48 h	BNZ	20 ± 2.1 **^a^	41.0 ± 1.4 ***^a^	47.0 ± 1.4 ***^a^
	F0.5v	0.0	5.66 ± 1.4 *^b^	59.4 ± 1.4 ***^b^
	AgFuc	0.6 ± 1.0	51.0 ± 3.1 ***^c^	67.3 ± 2.1 ***^c^
	Ag	7.6 ± 1.7 *^b^	13.2 ± 1.8 **^d^	25.8 ± 2.1 **^d^
	F0.5v:Ag (9:1)	0.0	4.3 ± 1.5 *^b^	55.4 ± 2.4 ***^b^
	Control #	0.0	0.0	0.0

# Control corresponds to medium without any sample. *p* < 0.05 (*); *p* < 0.01 (**); *p* < 0.001 (***) compared to the control group. ^a,b,c,d^ Different letters indicate a significant difference between each sample at the same concentration (*p* < 0.01). Three independent experiments were carried out (*n* = 5). BNZ—benznidazole. Ag = silver. F0.5v:Ag—a mix of silver and F0.5v at the same concentration that found in the AgFuc (9 parts of F0.5v and 1 part of Ag).

## Data Availability

Not applicable.
